# Utilizing Lactic Acid Bacteria to Improve Hyperlipidemia: A Comprehensive Analysis from Gut Microbiota to Metabolic Pathways

**DOI:** 10.3390/foods13244058

**Published:** 2024-12-16

**Authors:** Changlu Ma, Chen Xu, Mumin Zheng, Shuwen Zhang, Qifeng Liu, Jiaping Lyu, Xiaoyang Pang, Yinghong Wang

**Affiliations:** 1 Department of Food and Bio-Engineering, Beijing Vocational College of Agriculture, Beijing 102442, China; machanglu@126.com; 2 Institute of Food Science and Technology, Chinese Academy of Agricultural Science, Beijing 100193, China; xu123chen456@163.com (C.X.); zmm1998vip@163.com (M.Z.); zhangshuwen@caas.cn (S.Z.); lvjiapingcaas@126.com (J.L.); 3 State Key Laboratory for Bioactive Substances and Functions of Natural Medicines and Beijing Key Laboratory of New Drug Mechanisms and Pharmacological Evaluation Study, Institute of Materia Medica, Chinese Academy of Medical Sciences and Peking Union Medical College, Beijing 100050, China; liuqifeng@wchscu.edu.cn

**Keywords:** hyperlipidemia, probiotics, *Lactobacillus casei* CAAS36, metabolomics, gut microbiota

## Abstract

Hyperlipidemia poses significant risks for cardiovascular diseases, with emerging evidence underscoring the critical role of gut microbiota in metabolic regulation. This study explores *Lactobacillus casei* CAAS36, a probiotic strain with promising cholesterol-lowering capabilities, assessing its impact on hyperlipidemic hamsters. Utilizing 1H NMR-based metabolomics and 16S rRNA gene sequencing, we observed that *L. casei* CAAS36 treatment not only altered metabolic pathways but also reshaped gut microbiota composition. Notably, the treatment restored the balance between Firmicutes and Bacteroidetes and significantly increased the abundance of propionate-producing Muribaculaceae. Metabolically, *L. casei* CAAS36 administration led to the normalization of key lipid markers, including reductions in total cholesterol, LDL-C, and triglycerides (29.9%, 29.4% and 32.6%), while enhancing the protective HDL-C levels. These effects were accompanied by significant increases in beneficial metabolites such as propionate and succinate, which are known for their roles in preventing metabolic disorders. These findings highlight the dual regulatory effects of *L. casei* CAAS36 on the metabolic profile and gut microbiota, suggesting a substantial potential for this probiotic in the management of hyperlipidemia and possibly other metabolic diseases. Future applications may include its use as a natural therapeutic agent in clinical settings, aiming to reduce reliance on conventional pharmaceuticals and their associated side effects.

## 1. Introduction

Hyperlipidemia, characterized by abnormally elevated levels of lipids or lipoproteins in the bloodstream [1], is increasingly recognized as a significant risk factor for cardiovascular diseases (CVD) [2,3,4], which are the leading cause of death globally [3]. Cardiovascular disease (CVD) is one of the leading causes of death worldwide [5]. According to the National Vital Statistics System of the National Center for Health Statistics (NCHS), 228,524 excess CVD deaths occurred from 2020 to 2022, which is 9% more CVD deaths than would have been expected based on trends from 2010 to 2019 [6]. Among these fatalities, a substantial proportion can be directly attributed to hyperlipidemia, due to its facilitation in the development of atherosclerosis and subsequent occurrences of heart attacks and strokes [4,7].

Currently, the common treatments for HLP are exercise, diet control, and medication [8]. High cost and limited accessibility limit the widespread use of drugs such as statins and proprotein convertase subtilisin/kexin type 9 (PCSK9) inhibitors [9]. Probiotics, particularly specific strains of Lactobacilli [10,11,12], present a promising strategy for the management of hyperlipidemia through modulating gut microbiota and systemic lipid levels [13]. This natural approach aligns with the increasing demand for sustainable healthcare interventions that minimize long-term pharmaceutical side effects.

However, the findings from various studies remain inconclusive. While certain strains of probiotics have demonstrated significant lipid-lowering effects [14,15], others have shown minimal or no negligible impact [16]. This variability can primarily be attributed to a limited understanding of the intricate mechanisms through which probiotics influence lipid metabolism. For example, a meta-analysis of randomized controlled trials indicated that while some strains effectively improved lipid profiles [17,18,19], others did not yield similar results [20], suggesting a complex interplay between specific bacterial strains and the host’s metabolic pathways.

The advent of the era of omics-based systems biology has opened up new horizons for understanding microbial–metabolite interactions [21,22]. In this study, we focus on *Lactobacillus casei* CAAS36, a strain isolated from homemade dairy products, which has shown initial success in reducing lipid levels in preclinical settings [23]. The primary objective of our research is to elucidate the mechanistic pathways through which *L. casei* CAAS36 exerts its effects, investigating its potential to modulate both gut microbiota and metabolic profiles. By providing a comprehensive analysis of these interactions, this work aims to solidify the foundation for using *L. casei* CAAS36 as a reliable natural therapy for hyperlipidemia. This research not only addresses an urgent need in hyperlipidemia management but also paves the way for broader applications in metabolic health, holding significant implications for global public health initiatives.

## 2. Materials and Methods

### 2.1. Strain Culture

The *Lactobacillus casei* strains 3, 18, SY13, AST18, and CAAS36 utilized in this experiment were isolated from dairy products produced by traditional herders’ families in Inner Mongolia, China. Meanwhile, the *L. casei* strains 30, 32, and 35 were obtained from infant fecal samples. The probiotic effects of these strains on lipid metabolism regulation in mice have been previously demonstrated [23]. Additionally, the *Lactobacillus rhamnosus* strain GG (ATCC 53101), serving as a control strain for cholesterol removal experiments, was procured from the China General Microbiological Culture Collection Center. *L. casei* and *L. rhamnosus* strains were cultured in de Man−Rogosa−Sharpe (MRS) medium under the conditions as described at 37 °C.

A fermentation tank was used to prepare bacterial powder on a large scale. The settings for the fermentation process were 37 °C, 200 rpm of stirring, and 14 h of fermentation. Following fermentation, the bacteria were collected by centrifugation. The bacteria were then resuspended in a 1:2 (volume: mass) ratio using the protective agent, which is made up of 11.7% skim milk powder, 7.8% trehalose, and 3.3% glycerol. The agent was sterilized at 115 °C for 20 min and allowed to cool to room temperature before use. After 24 h of freezing at −80 °C, it was vacuum-freeze-dried for 48 h and then stored at 4 °C. The viable bacterial count was determined by finite gradient dilution method using MRS agar (1.5% *w*/*v*) plates.

### 2.2. Reagents

The control diet employed in this study was procured from Beijing HFK Bioscience Co., Ltd., Beijing, China. To induce hyperlipidemia in hamsters, the high-fat diet (HFD) was prepared by supplementing the control diet with 20% lard oil and 0.2% cholesterol content (Supplementary Table S1). All reagents required for lipid profile assays, including total cholesterol [24], triglycerides (TG), low-density lipoprotein cholesterol (LDL-C), and high-density lipoprotein cholesterol (HDL-C), were purchased from BIOSINO Biotech. and Sci., China. Sodium thioglycollate, Oxgall, and a water-soluble form of cholesterol (polyoxyethanyl-cholesteryl sebacate) were sourced from Sigma-Aldrich, St. Louis, MO, USA. Fenofibrate, used as a comparative pharmaceutical agent, was obtained from Abbott Laboratories, Green Oaks, IL, USA.

### 2.3. Cholesterol-Lowering Activity

The cholesterol assimilation by the cultures was assessed using a modified version of the method described by Danielson et al. [25]. Fresh cultures of *L. casei* CAAS36 were inoculated at a concentration of 1% into MRS broth supplemented with 0.2% sodium thioglycollate and 100 mg/L cholesterol-PEG600 from Sigma-Aldrich, along with 0.3% (*w*/*v*) oxgall to simulate intestinal conditions. The cultures were incubated at 37 °C for a duration of 24 h. Post incubation, cells were separated through centrifugation, and the cholesterol levels in the spent broth were quantified using the o-phthalaldehyde method.

### 2.4. Animal Treatment

Hamsters are very similar to humans in the ratio of cholesterol to bile acids [26], the way bile acids are metabolized [27], and the diet-induced atherosclerotic damage. Specifically, the endogenous cholesterol synthesized in hamsters mainly occurs outside the liver, and this ratio is about 85%, which is similar to 90% in humans [28]. The liver of hamsters synthesizes bile acid and cholesterol at lower levels, and they induce cholesterol 7α-hydroxylase less, which makes hamsters more sensitive to high-cholesterol feed than rats, making it easier to establish a hyperlipidemia model [29]. In addition, golden hamsters have a liver-specific expression of cholesterol ester transfer protein (CETP) similar to that of humans. When cholesterol increases, CETP can transfer cholesterol from HDL to LDL. Therefore, when cholesterol increases, HDL decreases and LDL increases, while rats and mice do not have CETP genes [30]. To summarize, hamsters have become the preferred animal for experimental models of hyperlipidemia.

Thirty-two male golden hamsters aged 7–8 weeks and weighing 100–120 g were placed in an animal room for adaptive feeding for 1 week. Subsequently, they were randomly divided into 4 groups, each with 8 animals, marked as normal control group (C), high-fat model group (M), *L. casei* CAAS36-treated group (L), and fenofibrate positive drug control group (F). The golden hamsters were fed with a high-fat diet for modeling for two weeks. After the modeling was completed, the drugs were administered by gavage once a day for 8 consecutive weeks. Among them, groups C and M were given 2.5% sodium carboxymethylcellulose (CMC-Na), group L was given *L. casei* CAAS36 at a dose of 200 mg/kg (10^10^ CFU, a dosage that shown a greater probiotic effect in the initial experiments [23]), and group F was given fenofibrate at a dose of 50 mg/kg. All drugs were prepared with 2.5% CMC-Na and administered by gavage at a ratio of 1 mL/100 g body weight. During this period, the weight changed, blood lipid status and other health indicators of the golden hamsters were regularly evaluated.

After 8 weeks of administration, various physiological and biochemical indices were measured in hamsters. The blood sampling experiment was arranged in the morning to conform to the 24 h biological rhythm of blood lipids and reduce sampling errors. Before the measurement, the hamsters needed to fast for 20 h to ensure the fasting state. About 0.2 mL of blood was collected through the medial canthal vein of the eye, and the blood collection process was strictly controlled to be completed within 2 h to ensure the freshness and accuracy of the sample, and then the serum biochemical indicators were tested immediately. When collecting feces, in order to avoid microbial contamination, the mouse cage, toothpick, gauze, and 1.5 mL EP tube were sterilized by high temperature and high pressure, and the ice box was placed in the transfer window for ultraviolet irradiation for more than 30 min in advance to ensure the sterility of the experimental materials. The end-of-intestinal extrusion method was used to stress the mice and induce them to defecate. After the mice defecated, 2–3 fecal samples were carefully collected using sterile toothpicks. To avoid cross-contamination, a new sterile toothpick was replaced after each feces collection. The collected fecal samples were immediately placed in a sterilized 1.5 mL EP tube and quickly placed on an ice box for precooling, to maintain the activity and stability of the samples. Subsequently, the EP tube was promptly transferred to a −80 °C refrigerator for long-term storage in preparation for subsequent 16S rRNA sequencing analysis. For the mouse feces and urine samples required for metabolome measurement, they were collected using metabolic cages to ensure that the metabolites of the samples were not affected by external environmental factors. After the experiment, the golden hamsters were euthanized by an intraperitoneal injection of 0.4 mL of 1% pentobarbital, and blood was then collected through the abdominal artery and liver, muscle, and adipose tissue were anatomically collected for subsequent analysis.

### 2.5. Serum Biochemistry

At the conclusion of the treatment period, blood samples were collected for biochemical analysis. The serum levels of TC, TG, LDL-C, and HDL-C were measured using commercial kits according to the manufacturer’s instructions provided by BioSino Biotechnology and Science Inc., Beijing, China.

### 2.6. Metabonomic Analysis

For detailed metabolic profiling, 1H-NMR spectroscopy was employed. Serum, urine, and fecal samples were collected from control, HFD, and *L. casei* CAAS36-treated groups. The samples were prepared and analyzed following the methodology described by Li et al. [31]. To prepare serum samples, 500 μL of serum was centrifuged at 13,000× *g* using a 3 kDa Nanosep microcentrifuge unit. After centrifugation, 450 μL of filtrate was mixed with 50 μL of 0.9% sodium buffer (100% D_2_O, 5 mM DSS) and transferred to a 5 mm NMR tube. Additionally, a separate mixture was prepared by mixing 120 μL of 0.9% sodium buffer (D_2_O = 1:9 with 0.1% TSP) with 60 μL of serum and transferred to a 3 mm NMR tube for testing. The urine was centrifuged at 13,000× *g* for five minutes in order to prepare the samples. After that, 300 μL of the supernatant was removed and mixed with 300 μL of phosphate buffer (100% D_2_O, 0.2 M NaH_2_PO4/K_2_HPO_4_, 1 mM DSS, pH 7.4) in a 5 mm NMR tube for examination using a Bruker Avance III 500 MHz spectrometer. Multivariate analysis was conducted to discern the metabolic changes among the different groups.

### 2.7. Microbiota Analysis

To assess alterations in the composition of gut microbiota, 16S rRNA gene sequencing was conducted on fecal samples collected during the intervention period. Bacterial DNA was extracted using the TIANamp Stool DNA Kit (Tiangen, Beijing, China), and the V3-V4 functional region of the bacterial 16S rRNA gene was amplified using specific primer sequences (357F 5′-ACTCCTACGGGAGGCAGCAG-3′ and 806R 5′-GGACTACHVGGGTWTCTAAT-3′) and sequenced on the Illumina MiSeq platform, as described by Du et al. [32].

### 2.8. Statistics

The experiment was repeated three times, and the results were expressed as the mean value plus standard deviation. Statistical analysis of the data was performed using SPSS 17.0 software, with one-way analysis of variance employed to examine intergroup differences. Multiple comparisons were conducted using the Turkey method, and a *p*-value less than 0.05 indicated statistically significant variations between groups.

## 3. Results

### 3.1. Measurement of Pathological Characteristics

The cholesterol removal by 9 *L. casei* strains cultivated in MRS medium supplemented with 0.3% oxgall are shown in Table S2. All strains were able to assimilate cholesterol at varying degrees. Analysis of variance revealed significant variation (*p* < 0.05) in the percentage of cholesterol assimilation among different probiotics. The cholesterol assimilation ranged from 15% to 52% at 24 h of incubation. Notably, *L. casei* CAAS36, 32 and 18 demonstrated significantly (*p* < 0.05) higher cholesterol removal ability (44%, 39% and 39%, respectively) compared with the other strains at the same time point. These results are in accordance with previous studies’ reports (Table S2) [33,34]. Based on the determination of bile salt hydrolase activity, the effects of these three strains on the lipid metabolism of hyperlipidemic model hamsters (including serum metabolic levels, arteriosclerosis index, organ index, liver pathology, etc.), and the intervention effects on the intestinal microbial diversity of hyperlipidemic model hamsters, *L. casei* CAAS36 with the best cholesterol-lowering capabilities was selected for more in-depth mechanistic studies.

**Figure 1:**
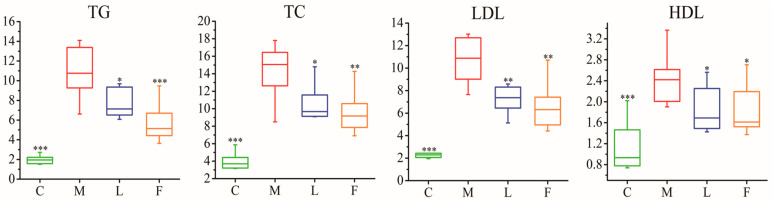
Box plots for effects of *L. casei* CAAS36 on serum TC, TG, LDL-C, and HDL-C levels in hyperlipidemia hamsters at week 8 after drug dosing. C: control group, M: model group, L: *L. casei* CAAS36- treated group, F: fenofibrate-treated group. The TG, TC, LDL, and HDL content in the model group was significantly increased compared with the control group, while the *L. casei* CAAS36-treated group was significantly decreased compared with the model group. Note: * *p* < 0.05, ** *p* < 0.01, *** *p* < 0.001, * indicates that each group is compared with the model group.

Serum biochemical parameters were measured at 8 weeks after dosage (Figure 1). In the HFD group, serum levels of TC, TG, LDL-C, and HDL-C significantly increased when compared with the control group, indicating that HFD increases the blood lipid levels in the hamsters. At this time, the *L. casei* CAAS36-treated group and fenofibrate-treated group significantly reduced the blood level of TC, TG, LDL-C, and HDL-C, and the lipid-lowering effects were initially shown. The above blood lipid index on one hand indicated that HFD successfully established a hyperlipidemia model; while on the other hand, *L. casei* CAAS36 and fenofibrate were effective in lowering blood lipid levels.

### 3.2. H NMR-Based Metabonomics Reveals Metabolic Changes in Hyperlipidemia Hamsters

**Figure 2:**
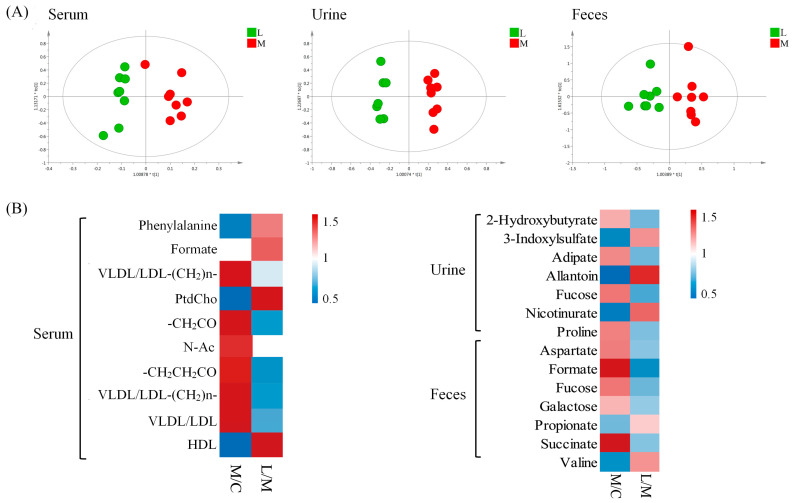
Metabolic changes in hyperlipidemic hamsters based on NMR metabolomics. (**A**) OPLS-DA score plots obtained from ^1^H NMR data of serum, urine, and fecal samples. (**B**) Potential biomarkers of serum and feces, metabolic pathways, as well as fold changes (M/C, L/M) between different groups at week 8. Note: C—control group, M—model group, L—*L. casei* CAAS36-treated group.

**Figure 3:**
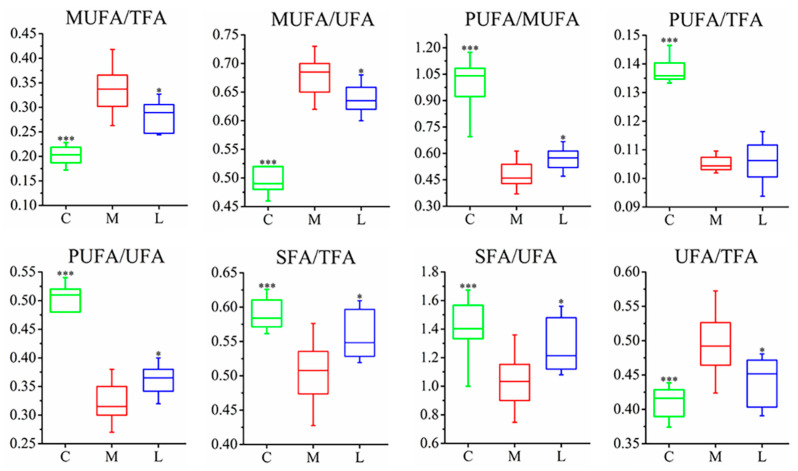
The ratio of fatty acid changes in different groups. C—control group, M—model group, L—*L. casei* CAAS36-treated group. The ratios of the model group are significantly changed compared with the control group, and the *L. casei* CAAS36-treated group can significantly restore these changes. Note: * *p* < 0.05, *** *p* < 0.001, * indicates that each group is compared with the model group.

Different groups of hamsters had their serum, urine, and fecal samples analyzed using 1H-NMR metabolomics. Significant differences were found in the metabolic profiles of the samples. The metabolite changes were evaluated using OPLS-DA to compare control, model, and *L. casei* CAAS36-treated groups. The quality of the model was evaluated as previously reported. According to the characteristics of metabolomics, the control group, hyperlipidemia group, and treatment group can be clearly distinguished. (Figure 2A, Figure S1). The corresponding OPLS-DA models all exhibited favorable qualities, with Q2 values ranging from 0.86 to 0.95, permutation tests, and CV-ANOVA results (Table S3). Metabolites that showed variable importance in *p* values less than 0.05 were selected as major metabolites (Figure 2B). The HFD model group showed significant differences from the normal group, similar to our previous report [31]. The analyses showed that *L. casei* CAAS36 treatments partly restored the levels of the changed metabolites by HFD-feeding. Specifically, we identified 11 restored metabolites in serum, 6 in urine, and 8 in feces (Figure 2B). These restored metabolites are involved in the lipid metabolism, tricarboxylic acid cycle (TCA) intermediate, carbohydrate metabolism, and amino acid metabolic pathways. Furthermore, we calculated the relative levels of saturated fatty acids (SFA) and unsaturated fatty acids (UFA), including monounsaturated fatty acids (MUFA) and polyunsaturated fatty acids (PUFA), in serum samples (Figure 3). The results showed that after treatment with *L. casei* CAAS36, except for no significant changes in PUFA/TFA indicators, all other fatty acid indicators were significantly improved, such as MUFA/TFA SFA/TFA, UFA/TFA, etc. The values of these indicators are closer to the control group compared to the model group.

### 3.3. Analysis of Differences in the Gut Microbiome Composition

**Figure 4:**
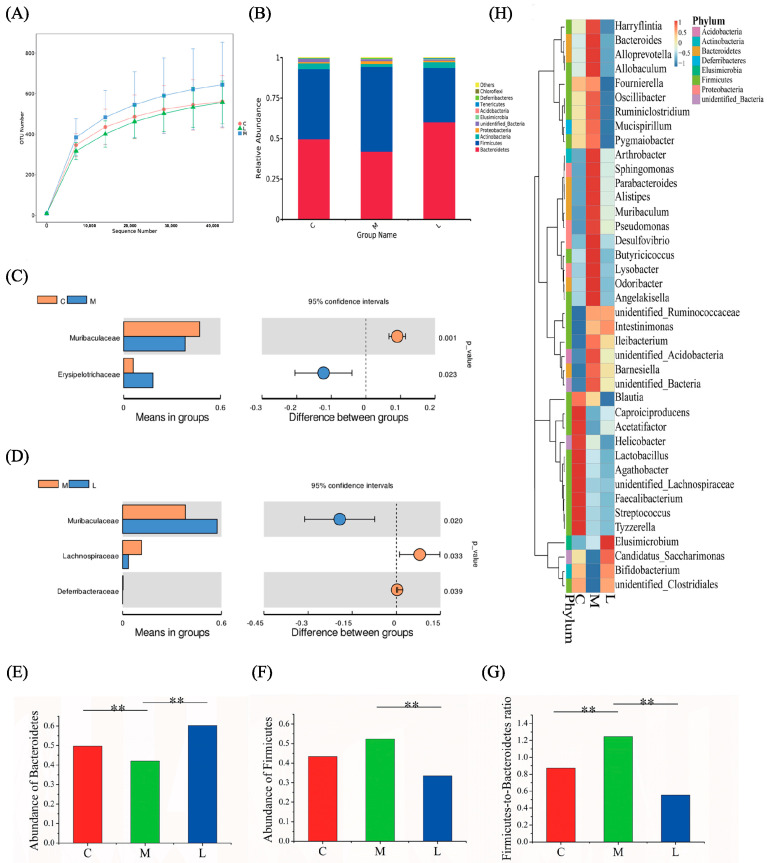
Intestinal flora analysis. (**A**) Evaluation of microbial richness and α−diversity in different groups based on Shannon index. The distribution and density of samples are displayed in box plot. Boxes represent the degree of dispersion (spread) and skewness in the data, the inside black plots represent the interquartile range, midline, range, mid-range, and trimean. *p* values are from Wil−coxon rank−sum test. (**B**) The relative abundance of intestinal microflora at the phylum level. The classification level is expressed as a percentage of the total sequence. The strips of different colors represent different taxa of intestinal microorganisms. (**C**,**D**) The significant gut microbial changes between control, model, and *L. casei* CAAS36 groups in family level. (**E**–**G**) Relative abundance of the Bacteroidetes (**E**) and Firmicutes (**F**) phyla, and the Firmicutes/Bacteroidetes ratio (**G**) in the control, model, and *L. casei* CAAS36 groups. (**H**) Relative abundance of the top 40 most different genera across groups. The abundance profiles are converted to Z-scores by subtracting the average abundance and dividing the standard deviation of all samples. The abundance profile is converted to a Z-score by subtracting the average abundance of all samples and dividing by the standard deviation. When the row abundance is lower than the average, the Z-Score is negative. Note: ** *p* < 0.01. Note: C—control group, M—model group, L—*L. casei* CAAS36-treated group.

To characterize the impact of *L. casei* CAAS36 on the gut microbiome, we conducted 16S rRNA gene sequencing analysis on fecal samples to assess microbial composition at the 8th week following HFD-model treatment. The bacterial 16S rRNA was extracted from fecal samples and sequenced on the IonS5TMXL platform, resulting in a total of 958,649 high-quality sequences with an average of 61,252 per sample. Rank Abundance curves were generated to compare OTU numbers between control, HFD-model, and *L. casei* CAAS36-treated groups. These curves exhibited smooth trends, sufficient when sequencing data were available, and only a minimal number of new OTUs remained undetected (Figure 4A).

At the phylum level, Firmicutes, Bacteroidetes, Actinobacteria, Proteobacteria, unidentified_Bacteria, Elusimicrobia, Acidobacteria, Tenericutes, Deferribacteres, and Chloroflexi (Top10) were identified as the dominant bacteria in the feces according to Figure 4B. Analysis of the family level revealed a decrease in the abundance of Muribaculaceae and an increase in the abundance of Erysipelotrichaceae in the HFD-model group (Figure 4C). In comparison to the HFD-model group, the *L. casei* CAAS36 treatment group showed lower abundances of Lachnospiraceae and higher abundances of Muribaculaceae and Deferribacteraceae (Figure 4D). The rest of the taxa results of t-test can be found in Figure S5. HFD-fed hamsters showed significantly increased abundance of the phylum Firmicutes, and a significantly reduced abundance of the phylum Bacteroides compared to controls; however, these changes were reversed by *L. casei* CAAS36 treatment. Furthermore, a greater proportion of Firmicutes to Bacteroidetes has been described in HFD relative to controls, and the change was reversed by *L. casei* CAAS36 (Figure 4E–G). The heat map displayed specific distribution patterns for each group based on the top 40 most different genera, as depicted in Figure 4H.

The sequences were assigned to a total of 22 different phyla. Among them, 15 phyla were consistently present in all groups (taxa present in >60% of rats per group, Figure S2), while only 4 phyla dominated with a relative abundance of ≥1%. The distribution and relative abundance of these dominant phyla varied among the HFD, *L. casei* CAAS36-treated groups, and the control group. Firmicutes, Bacteroidetes, Actinobacteria, and Proteobacteria were the predominant phyla that governed the composition of gut microbiota across different groups (Figure S2, Table S4), which aligns with the previous report [31]. In total, 142 families were identified from the sequence reads (Table S5). Muribaculaceae, Erysipelotrichaceae, Ruminococcaceae, Lactobacillaceae, Lachnospiraceae, Bifidobacteriaceae, Prevotellaceae, and Helicobacteraceae were the relatively abundant families among all groups, with a relative abundance of ≥1% (Figure S3). Moreover, 257 OTUs were classified into different genera (Table S6). All groups shared a core set of bacterial genera that were present at ≥1% of relative abundance in at least one group, including Ileibacterium, Lactobacillus, unidentified Ruminococcacea, unidentified Lachnospiraceae, Bifidobacterium, Allobaculum, and Helicobacter (Figure S4).

### 3.4. Correlation Between Metabolites and Intestinal Microbiota

**Figure 5:**
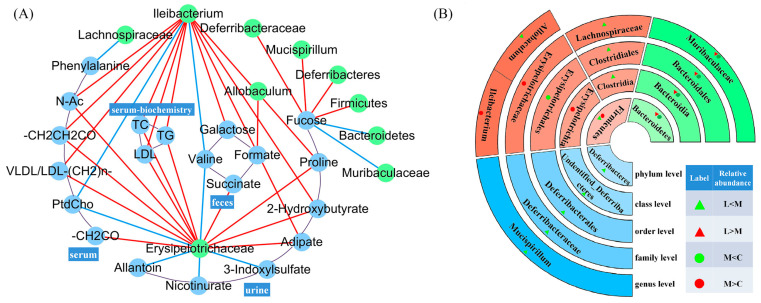
Correlation analysis between metabolites and intestinal flora in hyperlipidemic hamsters treated with *L. casei* CAAS36. (**A**) Correlation of abundance of significant changed bacterial taxa and metabolic parameters associated with obesity: red line for positive correlations and blue line for negative correlations. Only significant correlations (*p* < 0.05), are represented. (**B**) The relative abundance of gut microbiota with significant (*p* < 0.05) differences at different taxa. From the inside to the outside: phylum, class, order, family, genus. ○: the fold change in M/C; △: the fold change in L/M; red color: fold change > 1; green color: fold change < 1. Note: C—control, M—high fat diet, (L)— *L. casei* CAAS36-treated.

Correlations of significant changes in the bacterial abundance with measurements of metabolic parameters were performed on all hamsters (Figure 5). The significant metabolites of serum, urine, and feces and serum biochemistry were the parameters that were tested.

The abundance of both the taxa Erysipelotrichaceae and Ileibacterium exhibited significant correlations with most metabolic parameters. A positive correlation was observed with 11 and 10 of the parameters, while a negative correlation was shown with 5 and 2 of all parameters. Interestingly, valine in feces and PtdCho in serum both showed a negative correlation with Erysipelotrichaceae and Ileibacterium, and valine and PtdCho decreased in the HFD-fed model group. We also observed that fucose in urine showed a positive correlation with Firmicutes, Deferribacteres, Deferribacteraceae, and Mucispirillum and showed a negative correlation with Bacteroidetes and Muribaculaceae. The serum biochemistry (including TC, TG, and LDL) showed a positive correlation with Ileibacterium and Erysipelotrichaceae. And, we had also found that Ileibacterium and Erysipelotrichaceae showed a positive correlation with the lipid of serum such as VLDL/LDL, N-Ac and VLDL/LDL-(CH2)_n_-. For fecal metabolism, succinate had a positive correlation with Erysipelotrichaceae, galactose had a positive correlation with Ileibacterium, and formate had a positive correlation with Allobaculum. For urine metabolism, we observed that proline, adipate, and 2-hydroxybutyrate had positive correlations with Ileibacterium, Erysipelotrichaceae, and 3-indoxylsulfate; only nicotinurate and allantoin had negative correlations with Erysipelotrichaceae.

## 4. Discussion

In our comprehensive analysis of metabolic and microbial alterations induced by a high-fat diet (HFD) and remediated by *L. casei* CAAS36, we observed significant modulation in both metabolic profiles and gut microbiota structures. This study highlights the dual action of the probiotic in reversing HFD-induced dysbiosis and restoring metabolic homeostasis, underpinned by changes in short-chain fatty acid (SCFA) production and lipid metabolism. These findings align with recent research demonstrating similar metabolic and microbiota modulations by probiotic interventions in hyperlipidemic models [35]. The observed changes encompassed alterations in the lipid profiles (SFA, UFA, MUFA, PUFA, PtCho, cholesterol, triglycerides), TCA intermediate (succinate), carbohydrates (fucose, galactose), short-chain fatty acids (SCFAs), and amino acid (aspartate, proline, valine and Phenylalanine), as well as other metabolites including 2-Hydroxybutyrate, 3-Indoxysulfate, allantoin, and nicotinate. Furthermore, our study also demonstrated that *L. casei* CAAS36 obviously affected the pathways with common biological functions in a taxonomy of family and order in Firmicutes, Bacteroidetes, and Deferribacteres. The administration of *L. casei* CAAS36 reversed the rate of Firmicutes and Bacteroidetes which was increased by HFD, and specifically increased Muribaculaceae to produce propionate. The administration of *L. casei* CAAS36 notably reversed disruptions in metabolic pathways, particularly those associated with lipid metabolism, and positively shifted the gut microbiota balance towards health-promoting bacteria such as Muribaculaceae, known for their SCFA production. These results underscore the probiotic’s role in mitigating lipid disorders and enhancing gut health, which is crucial for metabolic regulation. Our findings are supported by similar observations in the literature, where probiotic treatment has led to improved lipid profiles and enhanced gut microbial diversity in metabolic syndrome contexts [34].

### 4.1. L. casei CAAS36 Treatment Induced Changes in Lipid Metabolism of HFD-Fed Hamsters

Our results showed that *L. casei* CAAS36 treatment decreased serum levels of TC, TG, and LDL-C, while these indexes increased significantly in HFD-fed hamsters (Figure 1). In this investigation, we noted marked increases in the N-Acetyl and VLDL/LDL ratios along with a reduction in serum phosphatidylcholine (PtCho) in the HFD group, indicative of significant lipid metabolic deregulation (Figure 3). These biomarkers reflect the disruptive impact of a high-fat diet on lipid homeostasis, which was effectively mitigated by *L. casei* CAAS36 treatment. The restoration of these parameters towards normal levels not only highlights the probiotic’s efficacy but also its potential mechanism of action through the modulation of lipid transport and inflammatory pathways. Such mechanisms are supported by Wang et al. [36], who described similar regulatory effects on lipid metabolism through probiotic administration. PtCho is the most important lipid in HDL and has a protective effect on diseases related to abnormal lipid metabolism [37]. Moreover, VLDL synthesis requires phospholipids such as PtCho. Therefore, the deficiency of PtCho or its precursors leads to reduced secretion of triglycerides by the liver, which leads to fatty liver. In the model group, we observed increased amounts of N-Acetyl glycoproteins (N-Ac), which is an essential marker of inflammation and protein reaction in acute phase [38]. After *L. casei* CAAS36 treatment, we observed increases in PtCho levels and decreased levels in the ratio of N-Ac and VLDL/LDL. Moreover, we observed a decreased PUFA/MUFA ratio that indicated the peroxidation of polyunsaturated fatty acids and oxidative stress. A previous study showed that MUFA exacerbates insulin resistance associated with obesity, while PUFA attenuates the level of insulin resistance [39]. After *L. casei* CAAS36-treatment, we observed increases in PUFA/MUFA ratio and decreased levels in the ratio of N-Ac and VLDL/LDL. The hyperlipidemic effect of several bacterial strains has been used in several animal models. Lactobacillus were able to increase in the ratio of HDL to LDL, and decreased triglyceride and cholesterol content as well as changing the gut microbiota in animals or rats fed a high-fat (HF) and high-cholesterol diet [40]. Our findings clearly demonstrate that *L. casei* CAAS36 not only ameliorates lipid metabolism disorders in HFD-fed animal models, but also orchestrates a broader regulatory role in metabolic functions. The probiotic treatment significantly lowered serum cholesterol and triglycerides, suggesting a systemic impact beyond the gastrointestinal tract, potentially involving signaling pathways linked to lipid metabolism. These observations are consistent with those of Yulianto et al. [41], who reported significant improvements in lipid profiles with probiotic interventions, highlighting the potential of targeted microbial therapies in managing dyslipidemia.

### 4.2. L. casei CAAS36 Treatment Induced Changes in SCFAs Metabolism of HFD-Fed Hamsters

In our previous study, the Lactobacillus strains were able to settle in the gut and ferment indigestible carbohydrates derived from foods. Their metabolic activities increase the amount of metabolites, such as SCFAs, which are mainly produced by carbohydrates, proteins, and peptides in the intestine [42]. They are able to block the synthesis of hepatic cholesterol, thus leading to a decrease in the amount of lipid in the blood [43].

**Figure 6:**
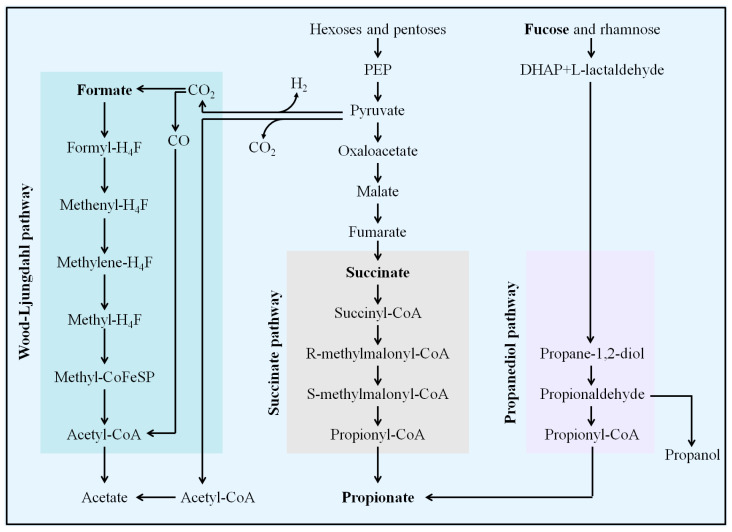
Synthesis of short chain fatty acids.

Dietary fiber is converted by microorganisms in the intestinal tract to produce three main SCFAs: acetate, propionate, and butyrate. Acetate is produced from pyruvate via acetyl-CoA and also via the Wood–Ljungdahl pathway. Formats are usually produced as by-products from the production of acetate via the Wood–Ljungdahl pathway. Propionate can be formed from phosphoenolpyruvate (PEP) through the succinate or acrylate pathway, in which lactate is reduced to propionate. Microorganisms can also produce propionic acid through deoxyhexose (such as fucose and rhamnose) through the propanediol pathway (Figure 6).

In this study, we discovered that HFD-feeding induced significant changes in SCFAs and Hexoses (fucose and galactose) for hyperlipidemia development in hamsters. These changes are associated with a decline of acetate in the serum and propionate in feces. Also, increasing formate, fucose, and galactose in the feces, and adipate in the urine were involved in the changes observed. After treatment with *L. casei* CAAS36, we observed the reversion in propionate, formate, fucose and galactose in feces (Figure 2B). Our results indicate that *L. casei* CAAS36 may be promoted to produce propionate through the succinate and propanediol pathways. It can also be verified by declined succinate, fucose, and galactose that are, respectively, intermediate products and substrates in these two pathways. Luminal acetate or propionate sensed by GPR41 and GPR43, releases PYY and GLP-1, affecting satiety and intestinal transit. Significantly, our study noted an increase in propionate production associated with *L. casei* CAAS36 administration, which could be implicated in metabolic benefits beyond lipid regulation. Propionate has been shown to be converted into glucose via gluconeogenesis, influencing satiety signals and reducing hepatic glucose output. This mechanism may contribute to the overall metabolic benefits observed, aligning with findings from Baggerman [44], who reported that dietary propionate supplementation leads to improved glucose homeostasis and insulin sensitivity in rodent models. A small amount of SCFAs (mostly acetate and possibly propionate) reach the circulation and can also directly affect the adipose tissue, brain, and liver, thus inducing overall beneficial metabolic effects [45].

### 4.3. L. casei CAAS36 Treatment Induced Changes in Proteolytic and Other Metabolites of HFD-Fed Hamsters

Intestinal microorganisms use SCFAs, that are the decomposite products of dietary fiber, as an energy source. However, when fermentable fiber is in short supply in HFD feeding, microorganisms turn to less favorable energy sources for growth, such as amino acids, endogenous proteins, or dietary fats from the diet. This results in a decrease in microbial community fermentation activity and concentration of products such as SCFAs. Protein fermentation can maintain the SCFAs pool [46]. Protein fermentative strains of the gut microbiota might be involved in mediating pro-inflammatory responses and dyslipidemia progression [47]. In particular, ammonia, phenolic compounds, indoles, hydrogen sulfide, and branched-chain fatty acids (BCFAs) have been associated with detrimental effects on gut epithelial health and gut permeability, as shown in rodent models fed with a HFD [48].

For instance, in our study, two reasons were responsible for the increase in adipate in the HFD group. Firstly, the fatty acids in the body were increased by the β oxidation rising in the high-fat diet, resulting in the inability of the body to produce enough carnitine or vitamin B2, thus leading to the degradation of adipate. Secondly, an increased production of branched-chain fatty acids during HFD feeding also caused an adipate increase in the HFD group. However, high levels of adipate can cause muscle and brain damage. Lower adipate in *L. casei* CAAS36 treatment group is likely due to the microbial reduction in amino acids, endogenous proteins, or dietary fats from the diet, because *L. casei* CAAS36 accelerates to ferment carbohydrates such as fucose and galactose.

HFD feeding also caused significant changes in fecal proteolyzes (Phenylalanine, Proline, Aspatate, valine), urinary BCFA metabolites (2-Hydroxybutyrate, adipate), and purine nucleotide metabolites (Nicotinurate, Allantoin). Also, *L. casei* CAAS36-treatment reversed the metabolic changes caused by hyperlipidemia.

### 4.4. L. casei CAAS36 Treatment Induced Changes in Gut Microbiota Structure of HFD-Fed Hamsters and Correlation of Metabolites

The levels of Firmicutes and Bacteroidetes divisions were found to be vastly different for obese and lean subjects in both animal models and the human cohort [37]. The Firmicutes to Bacteroidetes ratio in terms of their abundance was significantly higher in the HFD group (1.25) than in controls (0.87). In response to HFD feeding, we observed an increase in Firmicutes phylum, which was mainly due to the increase in abundance of Erysipelotrichaceae (Figure 5B). In the correlation analysis of gut microbiota and metabolites, the abundance of this family was positively correlated with the lipids of serum (VLDL/LDL, N-Ac and VLDL/LDL-(CH2) n-), succinate, proline, adipate, and negatively correlated with valine in feces, PtdCho in serum and 3-indoxylsulfate, Nicotinurate, and Allantoin in urine. This is consistent with the previously reported enrichment of Erysipelothrix in obese mice [49]. At the same time, the abundance of Muribaculaceae of the Bacteroidetes phylum was significantly reduced after HFD feeding, which was also the reason for the increase in F/B. After hyperlipidemic hamsters were treated with *L. casei* CAAS36, a decrease in the abundance of Firmicutes, represented by Ileibacterium, Allobaculum, and Lachnoclostridium, and a significant increase in the abundance of Bacteroidetes, represented by Muribaculaceae, were observed, reversing the Disturbance of intestinal flora caused by HFD (F/B = 0.55). Among them, Ileibacterium and Allobaculum belong to the Erysipelotrichaceae family. These responsive strains cope with the challenges of intestinal lipid metabolism posed by a high-fat diet via various effector networks.

Ileibacterium has been shown to synthesize non-classic amino acid conjugation of the bile acid cholic acid (AA-CA) [50]. Bile acid increases its water solubility by binding to amino acids, thereby better emulsifying lipids in the small intestine, which greatly enhances the lipolysis of pancreatic lipase at the fat–water interface and promotes lipid decomposition and absorption [51]. However, AA-CA has been shown to enhance Wnt signaling, which plays a key role in intestinal stem cell proliferation [50]. Under continuous lipid metabolic pressure, the abundance of Ileibacterium increases, which may lead to the excessive production of AA-CA, thereby abnormally activating the Wnt signaling pathway and promoting the development of colon cancer.

Lachnoclostridium often plays a negative role in a variety of diseases, such as serving as a fecal bacterial marker for the diagnosis of adenomas [52], being associated with intratumoral tertiary lymphoid structure (It-TLS) in patients with hepatocellular carcinoma [53], and producing trimethylamine to promote atherosclerosis [54]. This study found that the abundance of Lachnoclostridium increased in the HFD group, while *L. casei* treatment could significantly reverse this trend, and found that the abundance of this strain was significantly negatively correlated with serum phenylalanine content. Previous studies have identified microbial-derived phenylacetic acid as a contributor to steatosis progression, possibly by promoting hepatic lipid accumulation through increased branched-chain amino acid (BCAA) utilization [55]. Taking these findings together, we speculate that Lachnoclostridium may increase hepatic lipid accumulation by metabolizing phenylalanine to phenylacetic acid, while *L. casei* CAAS36 reduces the abundance of Lachnoclostridium in the intestine by competing for ecological niches, thereby reducing phenylacetic acid concentration and restoring phenylacetic acid and alanine levels, slowing the progression of hepatic steatosis.

In comparison to the control group, the abundance of Allobaculum in the HFD group was markedly increased. Significantly, following treatment with *L. casei*, the abundance of this microbiota exhibited a pronounced restoration. Multiple prior investigations have established that in mice and rats, the relative abundance of Allobaculum is significantly associated with the high-fat dietary patterns and fatty acid metabolic activities [56,57,58,59,60]. While investigating the mechanisms of lipid metabolism, the researchers revealed the dual role of ANGPTL4 (angiopoietin-like protein 4): in addition to being a central regulator of lipid metabolism [61,62,63], it also acts as a circulating mediator between the gut microbial community and fat deposition [64]. In a mouse model raised on a high-fat diet, the expression level of ANGPTL4 was positively correlated with the abundance of Allobaculum in the gut, suggesting that ANGPTL4 may modulate the abundance of microorganisms such as Allobaculum, which in turn affects fat deposition [65].

Short-chain fatty acid (SCFA) butyrate is a key factor in the regulation of host fat storage [66]. In the present study, we observed a significant increase in both butyrate levels and abundance of the gut microbial Allobaculum in the high-fat diet (HFD) group. This finding suggests that the increase in Allobaculum under high-fat diet conditions may be related to its butyrate-producing capacity, which may have an impact on fat storage. According to Janssen et al., the abundance of butyrate-producing Allobaculum in the intestine was significantly reduced in the ANGPTL4 knockout mouse model compared to wild-type mice [67]. This phenomenon supports the hypothesis that ANGPTL4 may influence host fat storage by modulating the intestinal abundance of butyric acid-producing microorganisms, particularly Allobaculum.

In addition, butyric acid has been shown to activate the peroxisome proliferator-activated receptor γ (PPARγ) in colonocytes and further regulate PPARγ target genes, including ANGPTL4 [68]. These findings reveal a possible network of interactions between the gut microorganism Allobaculum, its metabolite butyric acid, and ANGPTL4. This network may be involved in the regulation of host lipid metabolism and energy homeostasis. Based on this interaction network, it is conceivable that regulating the expression of ANGPTL4 may affect the abundance of Allobaculum and the production of butyric acid, which in turn affects host lipid storage and energy homeostasis, providing new perspectives for the development of new therapeutics targeting metabolic diseases such as obesity and diabetes.

In previous studies, the abundance of Muribaculaceae in Bacteroidetes was closely related to propionate [35]. This is consistent with our results, where the abundance of Muribaculaceae in Firmicutes by HFD feeding was decreased and increased in the *L. casei* CAAS36-treatement group. The negative correlation between the Muribaculaceae family and inflammatory bowel disease, obesity, and type 2 diabetes has been widely confirmed [69]. Combined with our results, the role of this bacterium in maintaining intestinal health may be found in the following two aspects. Muribaculaceae can produce short-chain fatty acids (SCFAs) such as acetate, propionate, and butyrate using endogenous (mucin polysaccharides) and exogenous polysaccharides (dietary fiber) [69]. SCFAs can affect the metabolism of adipocytes and intestinal epithelial cells through G protein-coupled receptors (such as GPR41 and GPR43), thereby regulating lipid metabolism [70]. At the same time, the increased abundance of Muribaculaceae is positively correlated with the activation of the PI3K/Akt signaling pathway [71], which plays a key role in regulating lipid metabolism and insulin sensitivity and can promote lipid storage and metabolism [72]. Therefore, Muribaculaceae may restore intestinal lipid metabolism through short-chain fatty acids and the PI3K/Akt signaling pathway. There are also reports that propionate reduces systemic inflammatory responses, atherosclerosis, and myocardial remodeling caused by hypertension through regulatory T cell balance [73] and the Muribaculaceae family that can produce propionate colonizes the mucus layer and is a symbiotic user of mucus polysaccharides in the intestine [74]. The increase in Muribaculaceae is associated with improved intestinal barrier function [75]. Improved intestinal barrier function can reduce intestinal permeability and endotoxemia, thereby reducing systemic inflammation and metabolic disorders [76], indicating that Muribaculaceae may repair intestinal inflammatory responses under high lipid metabolic stress by reducing inflammation and improving intestinal barrier function.

In addition, the abundance of Mucispirillum genera of Deferribacteres phylum was positively correlated with the fucose in feces. *Mucispirillum schaedleri* has been found in the intestines of some rodents and is thought to be associated with diseases. *Mucispirillum schaedleri* is commonly found in the intestinal mucous layer of rodents and other animals, and is considered to be a morbid symbiont, which plays a role in diseases. Previous studies have shown that *Mucispirillum schaedleri* can modify gene expression in mucosal tissue, indicating an intimate interaction with its host and a possible role in inflammation [77].

The results of our research showed that *L. casei* CAAS36 not only reversed the changes in metabolites, but was also beneficial in maintaining the structure of the gut microbiota. The administration of *L. casei* CAAS36 effectively countered the dysbiosis induced by HFD feeding, as evidenced by the restoration of the Firmicutes to Bacteroidetes ratio and a reduction in the abundance of pathogenic bacteria such as Mucispirillum. Concurrently, there was an increase in beneficial Muribaculaceae, associated with improved gut health and metabolic functions. This probiotic-induced microbial rebalance is critical, as highlighted in recent research by Lee [78] which links such bacterial shifts with enhanced metabolic resilience and reduced inflammation in hyperlipidemic conditions. In our study, the administration of *L. casei* CAAS36 enriched health-promoting bacteria capable of fermenting dietary carbohydrates to produce metabolically active compounds like propionate and succinate. These short-chain fatty acids (SCFAs) play a pivotal role not only in lipid metabolism but also in modulating host–microbe signaling pathways that influence colonic pH, microbiota composition, gut motility, and epithelial cell proliferation. This holistic influence underscores the therapeutic potential of probiotics in managing hyperlipidemia and its associated conditions. Parallel findings by Chen et al. [79], which illustrate how SCFAs from probiotic fermentation can enhance the intestinal barrier function and systemic immune response, further support our observations.

Although 1H-NMR can detect all chemical components in the sample without structure and polarity bias, which is suitable for non-target overall metabolic profile analysis, it does not have a chromatographic separation function and has a narrow dynamic range, resulting in low sensitivity and resolution, which may lead to detection limitations of low-concentration metabolites and quantitative analysis limitations. In future studies, we can explore the evaluation of the cholesterol-lowering ability of strains using multiple concentration gradients, improve dose optimization, and follow up on the long-term effects of *L. casei* CAAS36 on hyperlipidemic hamsters so as to provide more precise experimental guidance for clinical experiments. In addition, in this study, there are some mechanisms of changes in strain abundance that cannot be explained due to insufficient research, such as (1) the effect of increased abundance of Melainabacteria on hyperlipidemic hamsters and (2) the mechanism by which probiotic treatment leads to a significant increase in the abundance of Rhizobiaceae, which may arise as symbiotic nitrogen-fixing bacteria in nitrogen-poor animals.

## 5. Conclusions

In summary, *L. casei* CAAS36 improves metabolism related to lipid, carbohydrate, Proteolytic and other metabolites in hyperlipidemic hamsters. In addition, the study on the correlation between characteristic metabolites and different biological indicators is helpful to further understand the metabolic network. In this study, we studied the correlation between the changes in intestinal flora and the composition of metabolites. The outcomes of our research indicate that *L. casei* CAAS36 significantly corrects metabolic dysregulation and restores a healthy intestinal microflora in hyperlipidemic hamsters, pointing to its robust efficacy across multiple physiological systems. This probiotic strain not only improves lipid profiles but also fosters a gut environment conducive to overall health, as evidenced by the normalization of microbial diversity and function.

## Data Availability

The original contributions presented in the study are included in the article and Supplementary Material, further inquiries can be directed to the corresponding author.

## Supplementary Materials

The following supporting information can be downloaded at: https://www.mdpi.com/article/10.3390/foods13244058/s1, Figure S1: OPLS-DA score plots derived from the 1H NMR data for serum, urine and feces samples. C: control group, M: model group, Figure S2: Relative abundance expressed as a percentage of the phylum that were dominant and altered with the grug treatment in different groups, Figure S3: Relative abundance expressed as a percentage of the family that were dominant and altered with the grug treatment in different groups, Figure S4: Relative abundance expressed as a percentage of the genera that were dominant and altered with the grug treatment in different groups, Figure S5: The significant gut microbial changes between control, model and *L. casei* CAAS36, Table S1: Diet composition, Table S2: The cholesterol removal (%) of the 9 probiotic bacteria, Table S3: Results of permutation tests and CV-ANOVA, Table S4: Relative abundance expressed as a percentage of the family that were dominant and altered with the grug treatment in different groups, Table S5: Relative abundance expressed as a percentage of the family that were dominant and altered with the grug treatment in different groups, Table S6: Relative abundance expressed as a percentage of the genera that were dominant and altered with the grug treatment in different groups.
