# Supplementary material for: Utilizing Lactic Acid Bacteria to Improve Hyperlipidemia: A Comprehensive Analysis from Gut Microbiota to Metabolic Pathways
Source: Foods. 2024 Dec 16;13(24):4058. doi: 10.3390/foods13244058 (PMC11675396; doi:10.3390/foods13244058)
Supplement: Supplementary file 1 [file foods-13-04058-s001.zip › foods-3294839-supplementary.pdf]

Supplementary Material

Utilizing Lactic Acid Bacteria to Improve Hyperlipidemia: A Comprehensive Analysis from Gut Microbiota to Metabolic Pathways

Changlu Ma [1,2,†], Chen Xu [2,†], Mumin Zheng [2], Shuwen Zhang [2], Qifeng Liu [3], Jiaping Lyu [2], Xiaoyang Pang [2,*] and Yinghong Wang [3,*]

[1] Department of Food and Bio-engineering, Beijing Vocational College of Agriculture, Beijing 102442, China; machanglu@126.com
[2] Institute of Food Science and Technology, Chinese Academy of Agricultural Science, Beijing 100193, China; pangxiaoyang@163.com
[3] State Key Laboratory for Bioactive Substances and Functions of Natural Medicines and Beijing Key Laboratory of New Drug Mechanisms and Pharmacological Evaluation Study, Institute of Materia Medica, Chinese Academy of Medical Sciences and Peking Union Medical College, Beijing 100050, China; wyh@imm.ac.cn
† Equal contribution: These authors contributed equally to this work.
* Correspondence: pangxiaoyang@163.com; wyh@imm.ac.cn

1. Supplementary Figures

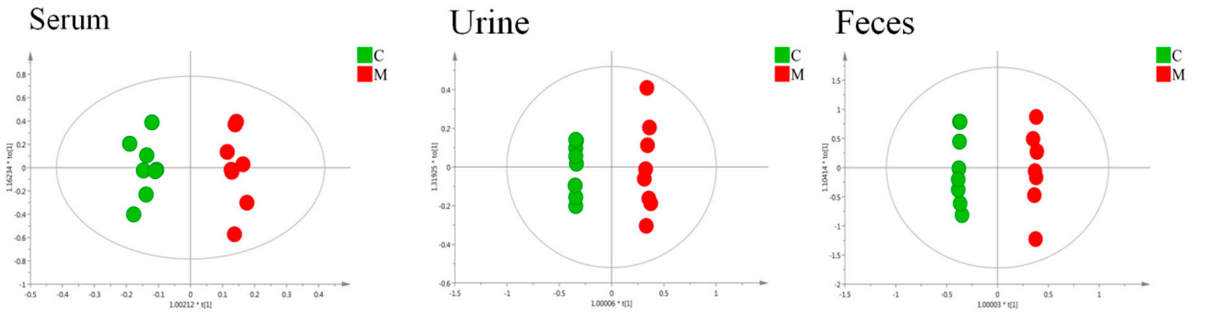

Figure S1. OPLS-DA score plots derived from the 1H NMR data for serum, urine and feces samples. C: control group, M: model group.

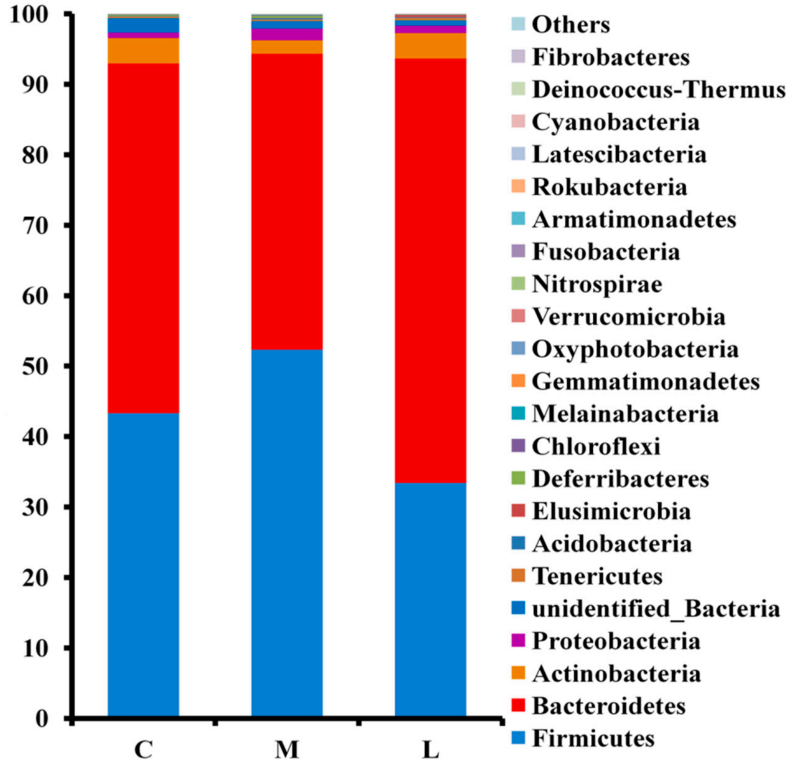

Figure S2. Relative abundance expressed as a percentage of the phylum that were dominant and altered with the grug treatment in different groups.

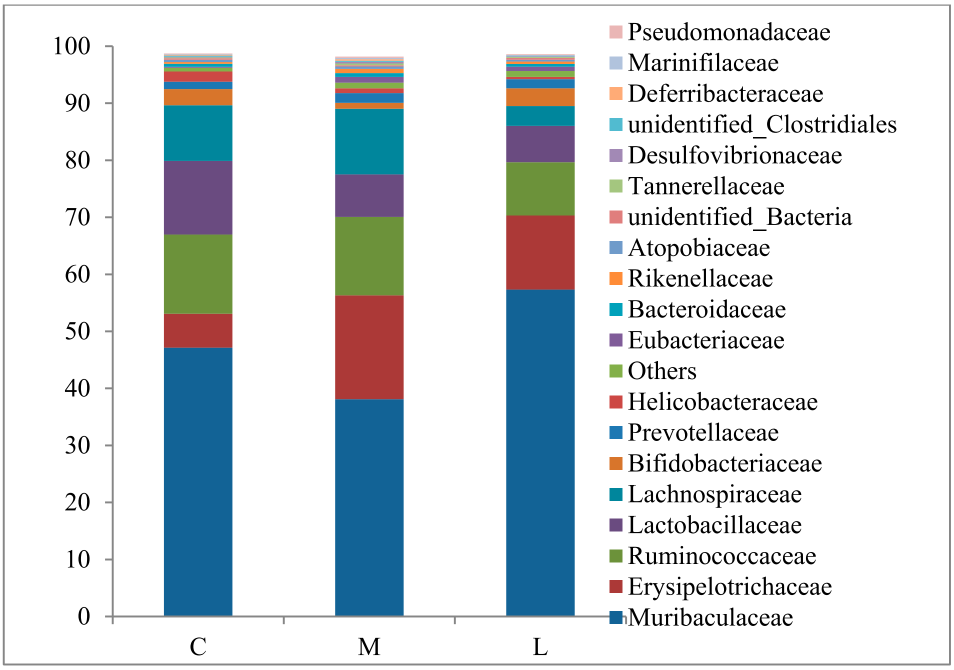

Figure S3. Relative abundance expressed as a percentage of the family that were dominant and altered with the grug treatment in different groups.

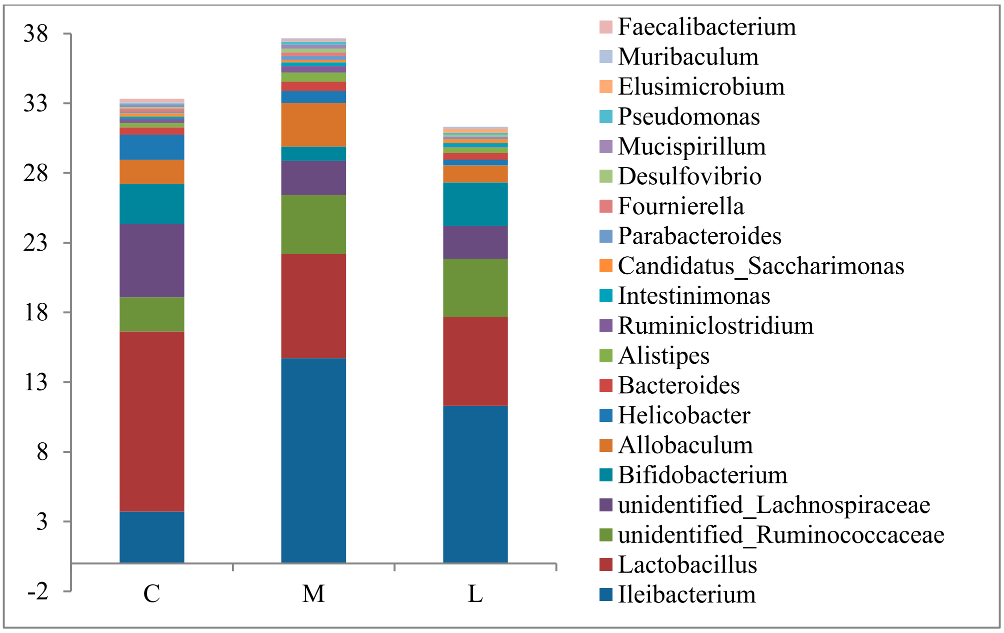

Figure S4. Relative abundance expressed as a percentage of the genera that were dominant and altered with the grug treatment in different groups.

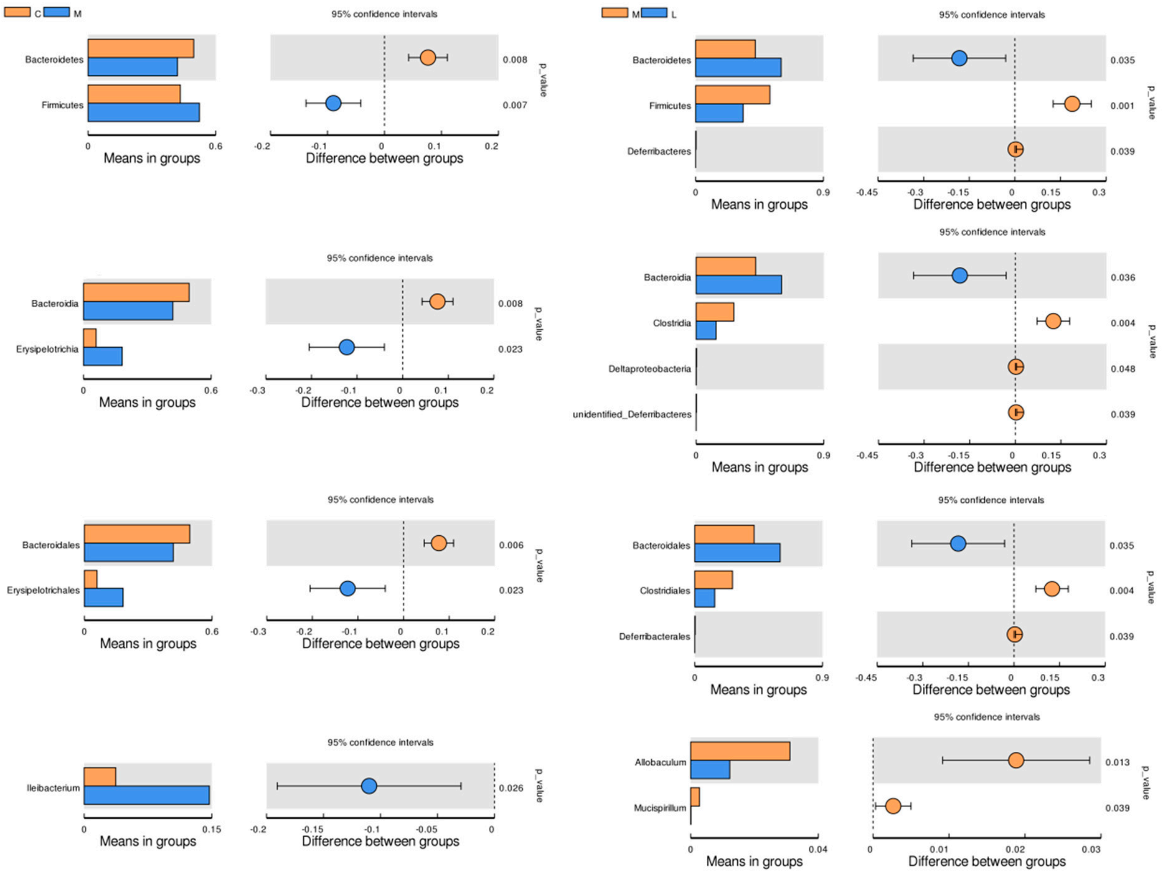

Figure S5. The significant gut microbial changes between control, model and L. casei CAAS36.

2. Supplementary Tables

Table S1. Diet composition

Ingredients	Control diet (CD, %)	High fat diet (HFD, %)
wheat middlings	19.2	15.3216
Bran	12.2	9.7356
Fishmeal	2.2	1.7556
Soybean meal	20.4	16.2792
Corn	40	31.9200
Soybean Oil	1.2	0.9576
Stone powder	1.52	1.21296
Calcium Hydrogen Phosphate	2	1.596
Salt	0.4	0.3192
Mineral salts	0.2	0.1596
L-methionine	0.2	0.1596
Choline	0.4	0.3192
Vitamins	0.08	0.06384
Lard	0	20
Cholesterol	0	0.2

Table S2. The cholesterol removal (%) of the 9 probiotic bacteria

Strain	Cholesterol removal （%）	Notes
L. casei CAAS36	44.28±4.45e	
L. casei 32	39.00±0.18de	
L. casei 18	38.85±0.68de	
L. casei 35	36.79±1.57cd	
L. casei 30	32.17±0.36bcd	
L. casei 3	31.59±3.70bcd	
L. casei AST18	30.06±1.97bc	
L. casei SY13	28.21±0.58 b	
L. rhamnosus GG	15.99±1.65a	As a reference strain

[1] Bacteria incubated in MRS supplemented with 100 mg/L water-soluble cholesterol and 0.3% oxgall for 24 h at 37 °C. Results are presented as the mean ±SD (n = 3).

Table S3. Results of permutation tests and CV-ANOVA.

Sample	Group	R2X	R2Y	Q2	CV-ANOVA p-value
Serum	M-C	0.782	0.972	0.783	0.032
	L-M	0.824	0.883	0.693	0.049
Urine	M-C	0.677	0.998	0.867	0.017
	L-M	0.790	0.986	0.849	0.048
Feces	M-C	0.863	0.999	0.968	0.004
	L-M	0.685	0.887	0.576	0.037

Table S4. Relative abundance expressed as a percentage of the family that were dominant and altered with the grug treatment in different groups.

Phyla	C	M	L
Firmicutes	43.328	52.343	33.411
Bacteroidetes	49.635	42.009	60.243
Actinobacteria	3.591	1.887	3.622
Proteobacteria	0.773	1.669	1.062
unidentified_Bacteria	2.075	1.069	0.738
Tenericutes	0.203	0.204	0.338
Acidobacteria	0.067	0.197	0.116
Elusimicrobia	0.004	0.055	0.298
Deferribacteres	0.181	0.280	0.012
Chloroflexi	0.046	0.076	0.053
Melainabacteria	0.016	0.045	0.037
Gemmatimonadetes	0.022	0.054	0.020
Oxyphotobacteria	0.014	0.018	0.007
Verrucomicrobia	0.007	0.027	0.009
Nitrospirae	0.001	0.012	0.004
Fusobacteria	0.021	0.008	0.007
Armatimonadetes	0.002	0.004	0.000
Rokubacteria	0.000	0.001	0.000
Latescibacteria	0.001	0.006	0.000
Cyanobacteria	0.000	0.002	0.000
Deinococcus-Thermus	0.002	0.000	0.002
Fibrobacteres	0.000	0.000	0.001
Others	0.012	0.034	0.022

Table S5. Relative abundance expressed as a percentage of the family that were dominant and altered with the grug treatment in different groups.

Family	C	M	L
Muribaculaceae	47.140	38.097	57.326
Erysipelotrichaceae	5.941	18.195	12.978
Ruminococcaceae	13.883	13.766	9.350
Lactobacillaceae	12.920	7.489	6.366
Lachnospiraceae	9.759	11.463	3.482
Bifidobacteriaceae	2.844	1.035	3.111
Prevotellaceae	1.290	1.734	1.578
Helicobacteraceae	1.804	0.856	0.409
Eubacteriaceae	0.189	1.025	0.764
Bacteroidaceae	0.527	0.683	0.474
Rikenellaceae	0.329	0.689	0.435
Atopobiaceae	0.390	0.451	0.294
unidentified_Bacteria	0.270	0.213	0.329
Tannerellaceae	0.117	0.295	0.171
Desulfovibrionaceae	0.112	0.321	0.128
unidentified_Clostridiales	0.215	0.183	0.108
Deferribacteraceae	0.181	0.280	0.012
Marinifilaceae	0.128	0.211	0.108
Pseudomonadaceae	0.085	0.203	0.132
Elusimicrobiaceae	0.004	0.053	0.298
Sphingomonadaceae	0.060	0.178	0.092
Burkholderiaceae	0.090	0.076	0.101
Eggerthellaceae	0.114	0.035	0.033
Xanthomonadaceae	0.035	0.088	0.050
Christensenellaceae	0.102	0.028	0.038
unidentified_Alphaproteobacteria	0.027	0.088	0.026
Hyphomicrobiaceae	0.042	0.042	0.049
Chitinophagaceae	0.030	0.065	0.028
unidentified_Rhizobiales	0.024	0.061	0.037
Peptococcaceae	0.037	0.055	0.028
Streptococcaceae	0.083	0.017	0.017
Barnesiellaceae	0.002	0.071	0.042
Enterobacteriaceae	0.031	0.009	0.074
Micrococcaceae	0.027	0.051	0.027
unidentified_Acidobacteria	0.012	0.054	0.033
Xanthobacteraceae	0.010	0.045	0.032
unidentified_Gammaproteobacteria	0.020	0.046	0.018
Rhodocyclaceae	0.017	0.045	0.020

unidentified_Acidimicrobiia	0.022	0.033	0.025
Rhizobiaceae	0.019	0.037	0.021
Nocardioidaceae	0.017	0.035	0.020
Gemmatimonadaceae	0.012	0.040	0.015
Pyrinomonadaceae	0.007	0.038	0.022
Microscillaceae	0.018	0.032	0.013
Rhodobacteraceae	0.012	0.025	0.014
Peptostreptococcaceae	0.043	0.006	0.001
Nitrosomonadaceae	0.007	0.030	0.011
Hyphomonadaceae	0.012	0.015	0.019
Cellvibrionaceae	0.012	0.022	0.009
Enterococcaceae	0.027	0.009	0.005
Caulobacteraceae	0.005	0.021	0.010
Saprospiraceae	0.003	0.026	0.006
Sphingobacteriaceae	0.009	0.017	0.006
Fusobacteriaceae	0.018	0.008	0.005
unidentified_Actinomarinales	0.007	0.012	0.012
Veillonellaceae	0.019	0.008	0.002
Gaiellaceae	0.005	0.013	0.008
unidentified_Dehalococcoidia	0.003	0.017	0.006
Corynebacteriaceae	0.019	0.001	0.004
Kineosporiaceae	0.005	0.014	0.003
Micromonosporaceae	0.008	0.009	0.004
Beijerinckiaceae	0.004	0.010	0.007
Geodermatophilaceae	0.007	0.009	0.005
Microbacteriaceae	0.005	0.011	0.005
Hymenobacteraceae	0.004	0.009	0.006
Caldilineaceae	0.005	0.007	0.007
Bdellovibrionaceae	0.008	0.006	0.003
Bacillaceae	0.007	0.007	0.002
Succinivibrionaceae	0.011	0.006	0.000
Akkermansiaceae	0.003	0.011	0.002
Nocardiaceae	0.015	0.000	0.002
Nitrospiraceae	0.001	0.012	0.004
Cellulomonadaceae	0.003	0.009	0.004
unidentified_Oxyphotobacteria	0.002	0.011	0.003
unidentified_Solibacterales	0.008	0.004	0.002
Leuconostocaceae	0.008	0.006	0.000
Streptomycetaceae	0.002	0.012	0.001
Actinomycetaceae	0.011	0.001	0.002
Iamiaceae	0.005	0.002	0.005

Rhodanobacteraceae	0.000	0.009	0.002
Haliangiaceae	0.001	0.007	0.002
Geminicoccaceae	0.000	0.007	0.002
Moraxellaceae	0.007	0.001	0.002
Alteromonadaceae	0.000	0.008	0.002
unidentified_Chloroflexi	0.002	0.002	0.004
Euzebyaceae	0.003	0.005	0.000
Cyclobacteriaceae	0.000	0.007	0.000
Pseudonocardiaceae	0.000	0.005	0.002
Carnobacteriaceae	0.005	0.002	0.001
Longimicrobiaceae	0.003	0.004	0.000
unidentified_Cardiobacteriales	0.005	0.002	0.000
Opitutaceae	0.000	0.005	0.002
Flavobacteriaceae	0.003	0.002	0.001
Staphylococcaceae	0.004	0.002	0.000
Archangiaceae	0.004	0.001	0.002
Roseiflexaceae	0.002	0.002	0.002
Solirubrobacteraceae	0.002	0.002	0.002
Rhodothermaceae	0.001	0.002	0.003
Rubritaleaceae	0.002	0.003	0.000
Solimonadaceae	0.002	0.002	0.002
Rickettsiaceae	0.003	0.002	0.001
Spirosomaceae	0.000	0.004	0.002
Anaeroplasmataceae	0.000	0.000	0.006
Chthoniobacteraceae	0.000	0.006	0.000
Acetobacteraceae	0.000	0.006	0.000
Legionellaceae	0.000	0.000	0.005
Woeseiaceae	0.001	0.002	0.002
unidentified_Melainabacteria	0.000	0.005	0.000
Propionibacteriaceae	0.005	0.000	0.000
Fimbriimonadaceae	0.002	0.002	0.000
unidentified_Rhodospirillales	0.004	0.000	0.000
Leptotrichiaceae	0.002	0.000	0.002
Parvularculaceae	0.000	0.003	0.001
Nannocystaceae	0.000	0.002	0.002
Balneolaceae	0.001	0.003	0.000
Paenibacillaceae	0.001	0.002	0.001
Mycobacteriaceae	0.002	0.000	0.000
Bacteriovoracaceae	0.000	0.002	0.000
Deinococcaceae	0.002	0.000	0.000
Polyangiaceae	0.001	0.002	0.000
Planococcaceae	0.001	0.002	0.000

Desulfuromonadaceae	0.002	0.001	0.000
Kiloniellaceae	0.001	0.002	0.000
Rhodospirillaceae	0.000	0.001	0.002
Syntrophaceae	0.000	0.001	0.002
Anaerolineaceae	0.002	0.001	0.000
Geobacteraceae	0.000	0.002	0.000
Methylophilaceae	0.000	0.002	0.000
Nostocaceae	0.000	0.002	0.000
Methylacidiphilaceae	0.000	0.000	0.002
Herpetosiphonaceae	0.000	0.002	0.000
Crocinitomicaceae	0.000	0.002	0.000
Trueperaceae	0.000	0.000	0.002
Aeromonadaceae	0.001	0.000	0.001
unidentified_Ignavibacteria	0.000	0.000	0.001
Thermomonosporaceae	0.000	0.001	0.000
Oligoflexaceae	0.001	0.000	0.000
Pasteurellaceae	0.001	0.000	0.000
unidentified_Deltaproteobacteria	0.001	0.000	0.000
Fibrobacteraceae	0.000	0.000	0.001
unidentified_Nostocales	0.000	0.001	0.000
Campylobacteraceae	0.001	0.000	0.000
Others	0.611	0.964	1.040

Table S6. Relative abundance expressed as a percentage of the genera that were dominant and altered with the grug treatment in different groups.

Genus	C	M	L
Ileibacterium	3.716	14.706	11.312
Lactobacillus	12.920	7.489	6.366
unidentified_Ruminococcaceae	2.440	4.208	4.165
unidentified_Lachnospiraceae	5.285	2.461	2.363
Bifidobacterium	2.844	1.035	3.111
Allobaculum	1.744	3.111	1.225
Helicobacter	1.804	0.856	0.409
Bacteroides	0.527	0.683	0.474
Alistipes	0.294	0.654	0.400
Ruminiclostridium	0.287	0.466	0.033
Intestinimonas	0.183	0.268	0.280
Candidatus_Saccharimonas	0.241	0.165	0.288
Parabacteroides	0.116	0.295	0.168
Fournierella	0.222	0.243	0.036
Desulfovibrio	0.091	0.278	0.109
Mucispirillum	0.181	0.280	0.012
Pseudomonas	0.083	0.198	0.129
Elusimicrobium	0.004	0.053	0.298
Muribaculum	0.079	0.163	0.108
Faecalibacterium	0.265	0.050	0.021
Oscillibacter	0.119	0.191	0.022
Odoribacter	0.091	0.151	0.084
unidentified_Clostridiales	0.090	0.027	0.089
Sphingomonas	0.030	0.114	0.058
Faecalibaculum	0.043	0.056	0.068
Blautia	0.088	0.071	0.003
Angelakisella	0.017	0.139	0.005
Pygmaiobacter	0.059	0.080	0.012
Agathobacter	0.110	0.019	0.007
Lachnoclostridium	0.054	0.056	0.009
Acetatifactor	0.079	0.009	0.031
Parasutterella	0.037	0.027	0.053
Barnesiella	0.002	0.071	0.042
Butyricimonas	0.032	0.060	0.022
unidentified_Bacteria	0.026	0.046	0.040
Butyricicoccus	0.015	0.084	0.002
Alloprevotella	0.029	0.052	0.017
Arthrobacter	0.027	0.046	0.027
Streptococcus	0.064	0.017	0.016

Harryflintia	0.028	0.062	0.003
unidentified_Acidobacteria	0.010	0.050	0.028
Caproiciproducens	0.071	0.000	0.012
Enterorhabdus	0.041	0.022	0.018
Lysobacter	0.019	0.041	0.014
Pedomicrobium	0.024	0.023	0.025
Ilumatobacter	0.022	0.029	0.022
Tyzzerella	0.045	0.012	0.009
Azoarcus	0.009	0.031	0.014
Pseudoxanthomonas	0.008	0.026	0.019
Marvinbryantia	0.027	0.016	0.005
Sphingobium	0.012	0.022	0.012
Rikenella	0.017	0.015	0.013
Devosia	0.010	0.021	0.014
Cellvibrio	0.012	0.022	0.009
Subdoligranulum	0.034	0.006	0.003
Terrimonas	0.009	0.023	0.010
Adlercreutzia	0.035	0.002	0.004
Enterococcus	0.027	0.009	0.005
Skermanella	0.007	0.025	0.009
Erysipelatoclostridium	0.021	0.007	0.012
Nocardioides	0.012	0.017	0.012
Acidibacter	0.005	0.027	0.008
Roseburia	0.017	0.016	0.005
Pelomonas	0.022	0.008	0.007
Novosphingobium	0.007	0.021	0.009
Anaerostipes	0.018	0.007	0.011
Arenimonas	0.007	0.017	0.012
Anaerofilum	0.012	0.019	0.002
Romboutsia	0.030	0.004	0.000
Gordonibacter	0.028	0.000	0.003
unidentified_Alphaproteobacteria	0.007	0.021	0.004
unidentified_Actinomarinales	0.007	0.012	0.012
Bauldia	0.009	0.015	0.007
unidentified_Erysipelotrichaceae	0.018	0.007	0.004
Nordella	0.005	0.014	0.010
Lachnospira	0.022	0.003	0.003
Altererythrobacter	0.005	0.015	0.007
Gaiella	0.005	0.013	0.008
Peptococcus	0.011	0.009	0.007
unidentified_Dehalococcoidia	0.003	0.017	0.006

unidentified_Gammaproteobacteria	0.009	0.009	0.007
Bilophila	0.006	0.015	0.005
Thauera	0.007	0.012	0.006
Hyphomicrobium	0.008	0.007	0.009
Reyranella	0.004	0.012	0.007
Fusobacterium	0.012	0.006	0.004
Marmoricola	0.005	0.011	0.006
Neorhizobium	0.008	0.009	0.004
Lactococcus	0.019	0.000	0.002
Dongia	0.006	0.012	0.002
Blastococcus	0.007	0.009	0.003
Oxalobacter	0.005	0.007	0.007
Bradyrhizobium	0.002	0.010	0.007
Steroidobacter	0.005	0.010	0.002
Bacillus	0.007	0.007	0.002
Succinivibrio	0.011	0.006	0.000
Akkermansia	0.003	0.011	0.002
Rhodococcus	0.015	0.000	0.002
Bdellovibrio	0.008	0.005	0.003
Cellulomonas	0.003	0.009	0.004
Phyllobacterium	0.008	0.004	0.004
Microvirga	0.002	0.008	0.006
unidentified_Oxyphotobacteria	0.002	0.011	0.003
Massilia	0.002	0.007	0.005
Intestinibacter	0.012	0.002	0.001
Megamonas	0.008	0.005	0.002
Weissella	0.008	0.006	0.000
Phenylobacterium	0.002	0.007	0.005
Streptomyces	0.002	0.012	0.001
Brevundimonas	0.002	0.008	0.003
Ferruginibacter	0.000	0.005	0.007
Rubellimicrobium	0.002	0.009	0.002
Pontibacter	0.001	0.006	0.006
Iamia	0.005	0.002	0.005
Chryseolinea	0.004	0.003	0.004
Haliangium	0.001	0.007	0.002
Hirschia	0.005	0.002	0.003
Anaerovorax	0.007	0.002	0.001
Azotobacter	0.002	0.005	0.003
Bryobacter	0.005	0.002	0.002
Rheinheimera	0.000	0.008	0.002
Stenotrophomonas	0.001	0.004	0.005
Sediminibacterium	0.009	0.000	0.000

unidentified_Chloroflexi	0.002	0.002	0.004
Cetobacterium	0.006	0.002	0.001
Actinomyces	0.007	0.001	0.001
Ascidiaceihabitans	0.007	0.002	0.000
Rhodomicrobium	0.000	0.005	0.003
Dialister	0.005	0.002	0.001
unidentified_Acidimicrobiia	0.001	0.004	0.003
Niastella	0.002	0.002	0.003
Virgisporangium	0.002	0.007	0.000
Sphingobacterium	0.004	0.003	0.001
Candidatus_Alysiosphaera	0.000	0.006	0.002
Rhodoplanes	0.000	0.006	0.002
Lechevalieria	0.000	0.005	0.002
Sphingopyxis	0.005	0.002	0.001
Candidatus_Entotheonella	0.003	0.002	0.002
Acinetobacter	0.005	0.000	0.002
Ignatzschineria	0.005	0.002	0.000
Kocuria	0.001	0.005	0.001
Sanguibacteroides	0.004	0.000	0.002
Azohydromonas	0.002	0.002	0.002
Turicibacter	0.002	0.002	0.002
Adhaeribacter	0.003	0.003	0.000
Archangium	0.004	0.001	0.002
Aeromicrobium	0.000	0.005	0.002
Acidovorax	0.004	0.000	0.002
Candidatus_Soleaferrea	0.000	0.007	0.000
Proteus	0.001	0.005	0.000
Solirubrobacter	0.002	0.002	0.002
Luteolibacter	0.002	0.003	0.000
Piscinibacter	0.002	0.002	0.002
Staphylococcus	0.004	0.002	0.000
Rickettsia	0.003	0.002	0.001
Noviherbaspirillum	0.003	0.000	0.002
Granulicatella	0.005	0.000	0.001
Delftia	0.002	0.003	0.000
Paraprevotella	0.003	0.002	0.000
Dyadobacter	0.000	0.004	0.002
Actinoplanes	0.002	0.000	0.004
Anaeroplasma	0.000	0.000	0.006
Algoriphagus	0.000	0.005	0.000
Legionella	0.000	0.000	0.005
Candidatus_Solibacter	0.003	0.002	0.000

Flavitalea	0.002	0.003	0.000
Woeseia	0.001	0.002	0.002
Polaromonas	0.001	0.002	0.002
unidentified_Rhizobiaceae	0.000	0.001	0.004
Defluviimonas	0.001	0.003	0.001
unidentified_Melainabacteria	0.000	0.005	0.000
Cutibacterium	0.005	0.000	0.000
unidentified_Rhodospirillales	0.004	0.000	0.000
unidentified_Nitrosomonadaceae	0.000	0.002	0.002
Veillonella	0.004	0.000	0.000
Holdemanella	0.004	0.000	0.000
Bosea	0.002	0.002	0.001
Sarcina	0.002	0.002	0.000
Amphiplicatus	0.000	0.003	0.001
Ammoniphilus	0.001	0.002	0.001
Eisenbergiella	0.000	0.000	0.004
Caenimonas	0.000	0.004	0.000
Pseudolabrys	0.000	0.002	0.002
Negativibacillus	0.002	0.002	0.000
Rhodovastum	0.000	0.003	0.000
Tahibacter	0.000	0.002	0.001
Tannerella	0.001	0.000	0.002
unidentified_Nitrospiraceae	0.001	0.002	0.000
Varibaculum	0.002	0.000	0.001
unidentified_Prevotellaceae	0.002	0.001	0.001
Caulobacter	0.000	0.002	0.000
Mycobacterium	0.002	0.000	0.000
unidentified_Chitinophagaceae	0.000	0.002	0.000
Comamonas	0.002	0.000	0.000
Roseomonas	0.000	0.002	0.000
Peredibacter	0.000	0.002	0.000
Capnocytophaga	0.002	0.000	0.000
Deinococcus	0.002	0.000	0.000
Catalinimonas	0.000	0.002	0.000
Anaerotruncus	0.001	0.002	0.000
Luedemannella	0.002	0.001	0.000
Planomicrobium	0.001	0.002	0.000
Opitutus	0.000	0.002	0.001
unidentified_Rhizobiales	0.000	0.002	0.001

unidentified_Syntrophaceae	0.000	0.001	0.002
Polycyclovorans	0.002	0.001	0.000
Lacunisphaera	0.000	0.002	0.001
Pelagibius	0.001	0.002	0.000
Megasphaera	0.002	0.001	0.000
Methyloceanibacter	0.000	0.002	0.001
Litorilinea	0.000	0.002	0.000
Segetibacter	0.001	0.001	0.001
Amaricoccus	0.001	0.001	0.001
Geobacter	0.000	0.002	0.000
Dubosiella	0.002	0.000	0.000
Alterococcus	0.000	0.002	0.000
Desulfuromonas	0.002	0.000	0.000
Herpetosiphon	0.000	0.002	0.000
unidentified_Methylacidiphilaceae	0.000	0.000	0.002
Chthonobacter	0.000	0.002	0.000
Taibaiella	0.000	0.002	0.000
Pseudoflavitalea	0.000	0.002	0.000
Fluviicola	0.000	0.002	0.000
Flavisolibacter	0.000	0.002	0.000
Phaeodactylibacter	0.000	0.002	0.000
Atopostipes	0.000	0.002	0.000
Ideonella	0.000	0.002	0.000
Truepera	0.000	0.000	0.002
Candidatus_Udaeobacter	0.000	0.002	0.000
Limnobacter	0.000	0.002	0.000
Flavobacterium	0.000	0.001	0.001
Labrys	0.000	0.001	0.001
Anaerofustis	0.000	0.001	0.001
Aeromonas	0.001	0.000	0.001
Enhydrobacter	0.001	0.001	0.000
Paracoccus	0.000	0.001	0.000
Chitinophaga	0.000	0.001	0.000
Variovorax	0.000	0.000	0.001
Sporacetigenium	0.001	0.000	0.000
Planktomarina	0.000	0.001	0.000
Azospirillum	0.001	0.000	0.000
Formosa	0.000	0.001	0.000
SUP05_cluster	0.001	0.000	0.000
unidentified_Ignavibacteria	0.000	0.000	0.001
Actinocorallia	0.000	0.001	0.000
Parasegetibacter	0.000	0.000	0.001

Haemophilus	0.001	0.000	0.000
Serratia	0.000	0.000	0.001
Oligoflexus	0.001	0.000	0.000
Pseudofulvimonas	0.000	0.001	0.000
unidentified_Deltaproteobacteria	0.001	0.000	0.000
Jeotgalicoccus	0.000	0.001	0.000
Sphaerotilus	0.000	0.000	0.001
unidentified_Nostocales	0.000	0.001	0.000
Campylobacter	0.001	0.000	0.000
